# REST-dependent glioma progression occurs independently of the repression of the long non-coding RNA *HAR1A*

**DOI:** 10.1371/journal.pone.0312237

**Published:** 2024-11-27

**Authors:** Ella Waters, Perla Pucci, Ruman Rahman, Anna P. Yatsyshyna, Harry Porter, Mark Hirst, Radka Gromnicova, Igor Kraev, Vera Mongiardini, Benedetto Grimaldi, Jon Golding, Helen L. Fillmore, Balázs Győrffy, Priyadarsini Gangadharannambiar, Christos N. Velanis, Christopher J. Heath, Francesco Crea

**Affiliations:** 1 School of Life, Health and Chemical Sciences, The Open University, Walton Hall, Milton Keynes, United Kingdom; 2 Division of Cellular and Molecular Pathology, Department of Pathology, University of Cambridge, Cambridge, United Kingdom; 3 School of Medicine, Biodiscovery Institute, University of Nottingham, Nottingham, United Kingdom; 4 Department of Human Genetics, Institute of Molecular Biology and Genetics of National Academy of Sciences of Ukraine, Kyiv, Ukraine; 5 Laboratory of Molecular Medicine, Istituto Italiano di Tecnologia, Genoa, Italy; 6 School of Pharmacy and Biomedical Sciences, University of Portsmouth, Portsmouth, United Kingdom; 7 Department of Bioinformatics, Semmelweis University, Budapest, Hungary; 8 Department of Biophysics, Medical School, University of Pecs, Pecs, Hungary; 9 Cancer Biomarker Research Group, Institute of Molecular Life Sciences, HUN-REN Research Centre for Natural Sciences, Budapest, Hungary; Tabriz University of Medical Sciences, ISLAMIC REPUBLIC OF IRAN

## Abstract

The long non-coding RNA (lncRNA), *HAR1A* is emerging as a putative tumour suppressor. In non-neoplastic brain cells, REST suppresses *HAR1A* expression. In gliomas REST acts as an oncogene and is a potential therapeutic target. It is therefore conceivable that REST promotes glioma progression by down-regulating *HAR1A*. To test this hypothesis, glioma clinical databases were analysed to study: (I) *HAR1A*/*REST* correlation; (II) *HAR1A* and *REST* prognostic role; (III) molecular pathways associated with these genes. *HAR1A* expression and subcellular localization were studied in glioblastoma and paediatric glioma cells. REST function was also studied in these cells, by observing the effects of gene silencing on: (I) *HAR1A* expression; (II) cancer cell proliferation, apoptosis, migration; (III) expression of neural differentiation genes. The same phenotypes (and cell morphology) were studied in *HAR1A* overexpressing cells. Our results show that *REST* and *HAR1A* are negatively correlated in gliomas. Higher *REST* expression predicts worse prognosis in low-grade gliomas (the opposite is true for *HAR1A*). *REST*-silencing induces *HAR1A* upregulation. *HAR1A* is primarily detected in the nucleus. *REST*-silencing dramatically reduces cell proliferation and induces apoptosis, but *HAR1A* overexpression has no major effect on investigated cell phenotypes. We also show that REST regulates the expression of neural differentiation genes and that its oncogenic function is primarily *HAR1A*-independent.

## Introduction

Gliomas are the most common primary brain tumours with very high mortality rates [1]. Their morphology can resemble that of different glial cells, including astrocytes, oligodendrocytes, and ependymal cells. This results in differing histologies, including lower grade I, II or III astrocytoma, oligodendroglioma, or the mixed cell oligoastrocytoma. Eighty percent of astrocytomas progress to the more aggressive grade 4 glioblastoma (GBM), which is scarcely differentiated. Patients diagnosed with GBM have a 5-year survival rate of less than 5%, which has not improved in decades [2]. Lower grade gliomas are the most frequent brain tumour in children, whereas GBM is a common brain tumour in adults [3]. Gliomas are highly infiltrative; therefore, surgery can rarely remove the whole tumour, and is not always an option depending on the location of the neoplasm (i.e., proximity to eloquent anatomical regions) and age of the patient. For example, Diffuse Midline Glioma (DMG), one of the most aggressive pediatric gliomas [4], is almost invariably inoperable. The characteristic inter- and intra-heterogeneity of gliomas makes developing therapeutic targets difficult [5]. Further research is required to identify earlier diagnostic markers, prognostic biomarkers for tumour progression and timing of recurrence and to identify more efficacious patient-tailored targeted therapeutics.

Long non-coding RNAs (lncRNAs) are transcripts longer than 200 nucleotides (nts), which do not encode proteins. LncRNAs have been implicated in multiple diseases, including cancer. Their expression is often specific to cellular location, tissue, and disease [6]. As lncRNAs are functional units, their subcellular localization is important to determine their function. Two understudied lncRNAs are the transcripts encoded by human accelerated region 1 A (*HAR1A*) and *HAR1B*. These lncRNAs overlap on human chromosome 13 and their sequences are human-specific [7]. *HAR1A* was shown to have a tumour suppressor role in oral cancer via interaction with Alpha Kinase-1 (ALPK1), and in non-small cell lung cancer via modulation of the Signal Transducers and Activator of Transcription 3 (STAT3) pathways [8, 9]. However, the function of these lncRNAs in gliomas has not been elucidated.

*HAR1A* and *HAR1B* transcription was shown to be repressed by RE1-silencing transcription factor (REST) in Huntington’s disease [10]. Intriguingly, REST is known to have an oncogenic role in gliomas, through the repression of tumour-suppressor genes. For example, REST downregulates synapsin-1 (SYN1), contributing to glioma pathogenesis [11, 12]. It is currently unknown whether the REST-*HAR1* axis is functionally relevant for glioma pathogenesis, or whether REST exerts its oncogenic function in a *HAR*-independent manner (e.g., primarily via the repression of protein-coding genes).

Based on this background, we hypothesize that REST exerts its oncogenic function by repressing HAR1A and HAR1B. Hence, the objectives of this study are to investigate the clinical significance of REST, HAR1A, and HAR1B expression in pediatric and adult gliomas and their functional roles in key cancer hallmarks (including cell survival, proliferation and migration).

## Materials and methods

### Bioinformatic analysis

#### CBioPortal

RNA-sequencing expression data of *HAR1A*, *HAR1B* and *REST* was analyzed on cBioPortal (https://www.cbioportal.org/, accessed on 4^th^ April 2023), to determine correlations in lower grade gliomas and glioblastoma. Data were obtained from Brain Lower Grade Glioma (TCGA, Firehose Legacy) and Glioblastoma Multiforme (TCGA, Firehose Legacy). Linear regression analysis was performed on GraphPad Prism 8 (GraphPad Software, San Diego, CA, USA). Sample size: the CBioPortal LGG study had 530 samples– 512 of these contained *HAR1A* data and 514 of these with *HAR1B* data; he GMB study had 619 samples– 157 of these had both *HAR1A* and *HAR1B*.

Additional analyses on the Pediatric CbioPortal (https://pedcbioportal.kidsfirstdrc.org/, dataset name: Pediatric Brain Tumor Atlas, accessed on the 25th of July 2023) were performed on the PBTA study (2182 samples).

#### Metascape

Gene ontology analysis of *HAR1A* and *HAR1B* was conducted on Metascape (https://metascape.org/, accessed on 4^th^ April 2023). Genes co-expressed with *HAR1A*, *HAR1B*, and *REST*, with an R^2^ threshold of ≥0.7 were selected from the cBioPortal lower grade glioma study, were queried on Metascape, and analyzed with ontology sources. The most significant regulators and most significant downstream pathways were analyzed on GraphPad Prism 8.

#### Kaplan-Meier plotter for correlation of prognosis (clinical datasets)

*HAR1A*, *HAR1B*, and *REST* expression correlation with prognosis in lower grade gliomas and glioblastoma were analyzed in the KM-plotter platform (https://kmplot.com), using the PAN-cancer Glioblastoma (N = 153) dataset and the PAN-cancer Lower Grade Glioma (N = 510) datasets. Genes co-expressed with *HAR1A* were selected from the Metascape analysis (R^2^ threshold of ≥0.7), and then analysed using the PAN-cancer Glioblastoma (N = 153) dataset on KM-plotter Private Edition (https://kmplot.com/private/). Genes significantly correlated with glioblastoma prognosis were further analyzed *in vitro*.

### Cell culture

U-373 MG (Uppsala) cells were obtained from the European Collection of Authenticated Cell Cultures (ECACC) and were cultured in EMEM media (ATCC) supplemented with a 1% (*v*/*v*) antibiotic-antimycotic solution and a 10% heat-inactivated fetal bovine serum (Thermo Fisher Scientific, Loughborough, UK). VUMC-DIPG-A (H3.3 K27M) cells were kindly provided by Dr. Esther Hulleman (VUMC Cancer Center, Amsterdam, the Netherlands), and were cultured in 1:1 DMEM-F12 and Neurobasal-A cell media and supplemented with a 10% heat-inactivated fetal bovine serum 1% (*v*/*v*) glutamax supplement, a 1% (*v*/*v*) antibiotic–antimycotic solution, 10 mM HEPES, a 1% (*v*/*v*) MEM non-essential amino acid solution, and 1 mM sodium pyruvate (Thermo Fisher Scientific), hereafter referred to as TBM medium. GCE28 and GIN28 cells were kindly provided by Dr. Ruman Rahman (University of Nottingham, Nottingham, UK), and were cultured with DMEM low glucose, supplemented with a 1% (*v*/*v*) antibiotic-antimycotic solution and a 10% heat-inactivated fetal bovine serum (Thermo Fisher Scientific). Cell lines were grown adherent and passaged using 0.25% (*v*/*v*) trypsin-EDTA and washed using HBSS (Thermo Fisher Scientific). LNCaP cells were obtained from ATCC and cultured in RPMI 1640 (GIBCO, Thermo Fisher) supplemented with 10% FBS and 1% penicillin-Streptomycin (Gibco, Thermo Fisher). Cells were incubated at 37°C and 5% CO_2_ in a humidified incubator.

### SiRNA reverse transfection

SiRNA knockdown of REST was performed using the reverse transfection method. Cells were seeded in 6-well plates (1.2 × 10^5^ cells/well) with a transfection mix containing RNAiMAX lipofectamine (Invitrogen, Thermo Fisher Scientific), siRNAs (IDT, Leuven, Belgium) and opti-MEM (Thermo Fisher Scientific). The final concentration of siRNA doses was 2 nM. The duplexes used were: anti-*REST* DsiRNA hs.Ri.REST.13.1 and hs.Ri.REST.13.2, and scrambled negative control DS NC1.

### Lentiviral overexpression

U-373 cells were stably transfected with a lentivirus-derived particle that induced *HAR1A* overexpression (Genecopoeia USA, Cat# LPP-CUST-GVO-GC). 7.0 × 10^4^ U-373 cells were seeded in 24-well plates and incubated overnight. The next day, fresh media was supplemented with a final concentration of 8 μg/ml polybrene (Sigma Aldrich, Gillingham, UK) to increase the efficiency of transduction. 5 μl of the purified human *HAR1A* lentiviral particles (Titer: 1.37 × 10^8^ TU/ml where 1 TU = 100 copies of viral genomic RNA) were added to the wells and incubated overnight. The next day, the wells were washed with media and incubated until confluent. 72 hours post-transduction, U-373 cells were split 1 in 2 into 6-well plates and were incubated to adhere. 0.3 μg/ml of puromycin (Sigma Aldrich) was used for antibiotic selection for 2 weeks, with media change every 3 days. These cells were referred to as U-373-HAR1A and results were normalized to cells transduced with the negative control vector, U-373-NC.

### Analysis of gene expression

Total RNA was isolated from cultured cells using the RNeasy Plus Mini Kit (Qiagen, Manchester, UK), according to the manufacturer’s instructions. Reverse transcription of 1 μg of RNA was performed using the High‐Capacity cDNA Reverse Transcription Kit (Applied Biosystems, Loughborough, UK), according to kit instructions. This cDNA was diluted 1 in 10 for RT-qPCR analysis. TaqMan gene expression assays were obtained from Thermo Fisher: *HAR1A* (Hs05038333_s1), *HAR1B* (Hs03299152_m1), *REST* (Hs05028212_s1), *GAPDH* (Hs02786624_g1), *MALAT1* (Hs00273907_s1), *HPRT1* (HS02800695_M1), *SNAP25* (Hs00938957_m1), *CPLX1* (Hs00362510_m1), *SYN1* (Hs00199577_m1), *DLG4* (Hs01555373_m1), *GABRG2* (Hs00168093_m1). *GAPDH* was used as the housekeeping gene, unless otherwise stated in the figure legends.

### Analysis of protein levels

Cell lysates were extracted using RIPA buffer. The protein was quantified using the Pierce BCA assay, according to the manufacturer’s protocol. Protein was resolved by gel electrophoresis on reducing SDS/PAGE. The proteins were transferred onto a nitrocellulose membrane. The membrane was then blocked in 5% non-fat milk dissolved in Tris-buffered saline (TBS). The blots were incubated overnight at 4°C with protein-specific antibodies. REST and GAPDH were the primary antibodies used. After the overnight incubation, the blots were washed in TBS with 0.1% Tween® 20 Detergent. The blots were then incubated with HRP-conjugated anti-rabbit secondary antibody for 1 hour at room temperature. The blot was then washed in TBS with 0.1% Tween^®^ 20. Finally, the blot was revealed by ECL substrate and visualized using Syngene Gbox with GeneTools software.

### Analysis of publicly available RNA-seq datasets

RNA-seq datasets from GIN31 and GCE31 cell lines (GSE233380) and MRI localised glioblastoma samples (GSE59612) were retrieved from the Gene Expression Omnibus (GEO). Data from primary and recurrent glioblastoma samples was retrieved from the TCGA-GBM cohort using the TCGAbiolinks (v2.31.3) package in R. Raw gene count data was processed in R (version 4.1.1) using RStudio (2021.09.0+351 Release). Differential expression analysis and variance stabilizing transformation was conducted using DESeq2 (v1.34.0). Differentially expressed genes were defined as those with an absolute log2 fold change greater than 1 and an adjusted p value less than 0.05.

### Localisation of lncRNAs

RNA was extracted from cells and fractionation was performed using the PARIS kit (Invitrogen), according to the manufacturer’s instructions. For RT-qPCR validation, the probes *MALAT1* was used as a nuclear marker, *GAPDH* as the cytoplasmic marker, and *HPRT1* as the housekeeping gene.

### Proliferation assays

U-373 and DIPGA cells were transfected with siRNAs, or the overexpression model plated and incubated for 2, 4, 6, and 8 days. On these days, the cells were trypsinised and a pellet was obtained to count the number of cells using the LUNA cell counter (Logos Biosystems). The number of cells were plotted using GraphPad Prism 8.

### Caspase 3/7 assays

1.0 × 10^4^ U-373 cells were plated on white 96-well pates and treated with siRNAs. On day 4 post-transfection, Caspase-Glo reagent (Promega, Southampton, UK) was added to each well, according to the manufacturer’s instructions. Luminescence was then quantified using the BMG POLARstar plate reader (BMG Labtech, Aylesbury, UK).

#### Wound healing assay (migration)

2.5 × 10^5^ *HAR1A*-overexpressed and control cells were plated in 24-well plates. Once confluent, a scratch was made in the cell monolayer using a sterile P20 pipette tip. The cells were imaged twice a day until the wound was closed. Images were analyzed using the MRI wound-healing tool on ImageJ.

### Statistical analysis

All data were obtained from two or three independent experiments, indicated in Figure legends by N, and analyzed using GraphPad Prism 8 software. Values presented as mean ± standard error of the mean (SEM). Significant differences between the groups were analyzed using one-way ANOVA with Dunnett’s multiple comparison test or two-way ANOVA with Šídák’s multiple comparison test. A p < 0.05 was set as threshold for statistical significance.

## Results

### Expression and clinical significance of REST, HAR1A and HAR1B in human gliomas

**Fig 1:**
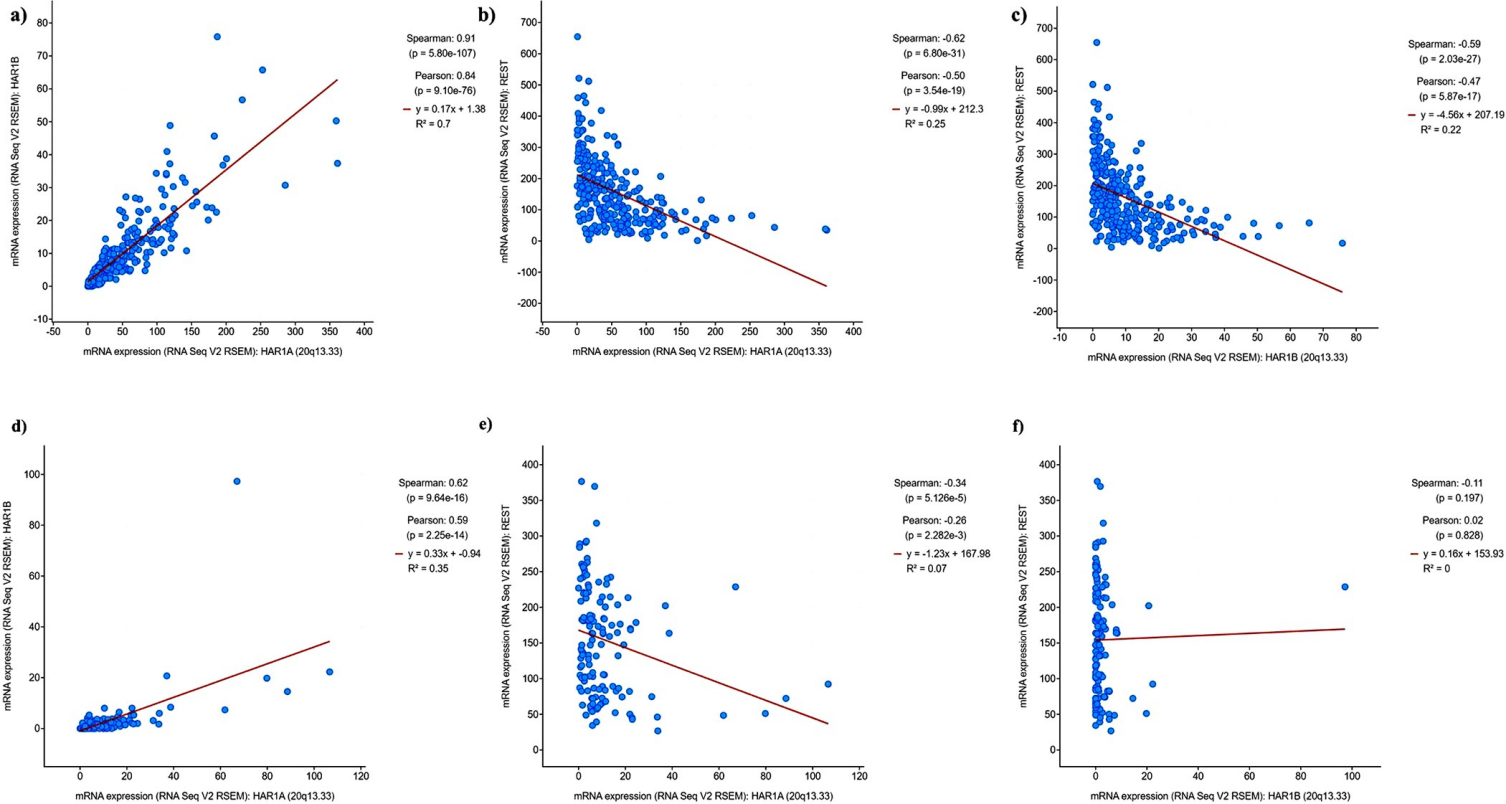
The expression and correlation of *HAR1A*, *HAR1B*, and *REST* in lower grade gliomas and glioblastoma. Linear regressions in lower grade gliomas of **a)** *HAR1A* and *HAR1B* (p<0.001, R^2^ 0.65), **b)** *HAR1A* and *REST* (p<0.001, R^2^ 0.2), **c)** *HAR1B* and *REST* (p<0.001, R^2^ 0.18). Linear regressions in glioblastoma of **d)** *HAR1A* and *HAR1B* (p<0.001, R^2^ 0.29), **e)** *HAR1A* and *REST* (p<0.001, R^2^ 0.07), **f)** *HAR1B* and *REST*. Expression z-score relative to all samples.

To analyze the relationship between *HAR1A* and *HAR1B* with *REST* in gliomas, we began by correlating their RNA expression in lower grade gliomas and glioblastoma, using the Cbio Portal. Linear regressions were analyzed in the following datasets: Brain Lower Grade Glioma (TCGA, Firehose Legacy) and Glioblastoma Multiforme (TCGA, Firehose Legacy) (Fig 1). We found that *HAR1A* and *HAR1B* have a significant positive correlation in both datasets (Fig 1A and 1D), *HAR1A* and *REST* have a significant negative correlation in both datasets (Fig 1B and 1E), and *HAR1B* and *REST* have a significant negative correlation in lower grade gliomas (Fig 1C), but unexpectedly, do not correlate in the GBM dataset (Fig 1F).

**Fig 2:**
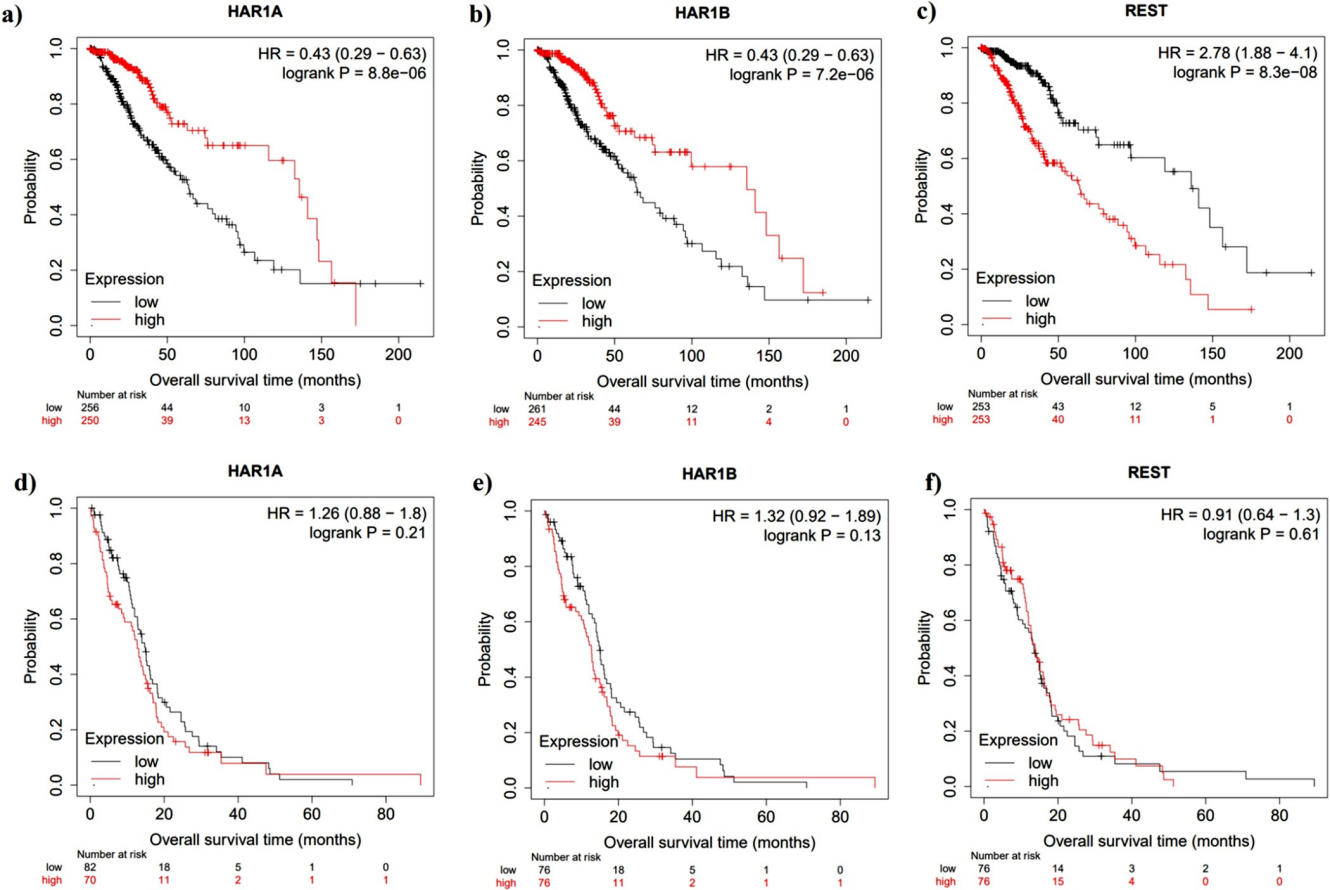
The overall patient survival of lower grade glioma and GBM patients, based on the expression of *HAR1A*, *HAR1B*, and *REST*. Kaplan-Meier plots from KMP-Plotter show high expression (red) or low expression (grey), relative to the median. Lower grade glioma: **a)** low *HAR1A* expression correlates with significantly worse lower grade glioma patient survival; **b)** low HAR1B expression correlates with significantly worse lower grade glioma patient survival; **c)** high REST expression correlates with worse lower grade glioma patient survival. Glioblastoma: **d)** *HAR1A*; **e)** *HAR1B*; **f)** *REST* expression does not correlate with glioblastoma patient prognosis.

Following the linear regression analysis of *HAR1A*, *HAR1B*, and *REST*, we determined whether aberrant expression of these lncRNAs and *REST* impacted glioma patient prognosis, using a Kaplan Meier analysis (Fig 2). In the lower grade glioma dataset, we found that a poorer prognosis of patients correlated with low expression of *HAR1A* and *HAR1B* (Fig 2A and 2B), and with high expression of *REST* (Fig 2C). In the GBM dataset, we found no significant correlation between patient overall survival for the lncRNAs or *REST* (Fig 2D–2F).

To test whether our results were also valid for pediatric malignancies, we performed correlation and survival analyses on a large pediatric glioma dataset (Pediatric CbioPortal, PBTA study). Our results confirmed that *HAR1A* and *HAR1B* are negatively correlated with *REST* expression in pediatric gliomas (S1A–S1C Fig). Interestingly, we found that none of the investigated genes had a prognostic significance in this dataset (S1D–S1F Fig).

Taken together, these results suggest that *HAR1A* and REST are negatively correlated in all glioma subtypes analyzed, and that the expression of these molecules is of prognostic relevance in adult lower grade gliomas, but not in the most aggressive gliomas (DIPG and GBM).

### Upstream and downstream pathways associated with HAR1A and HAR1B

**Fig 3:**
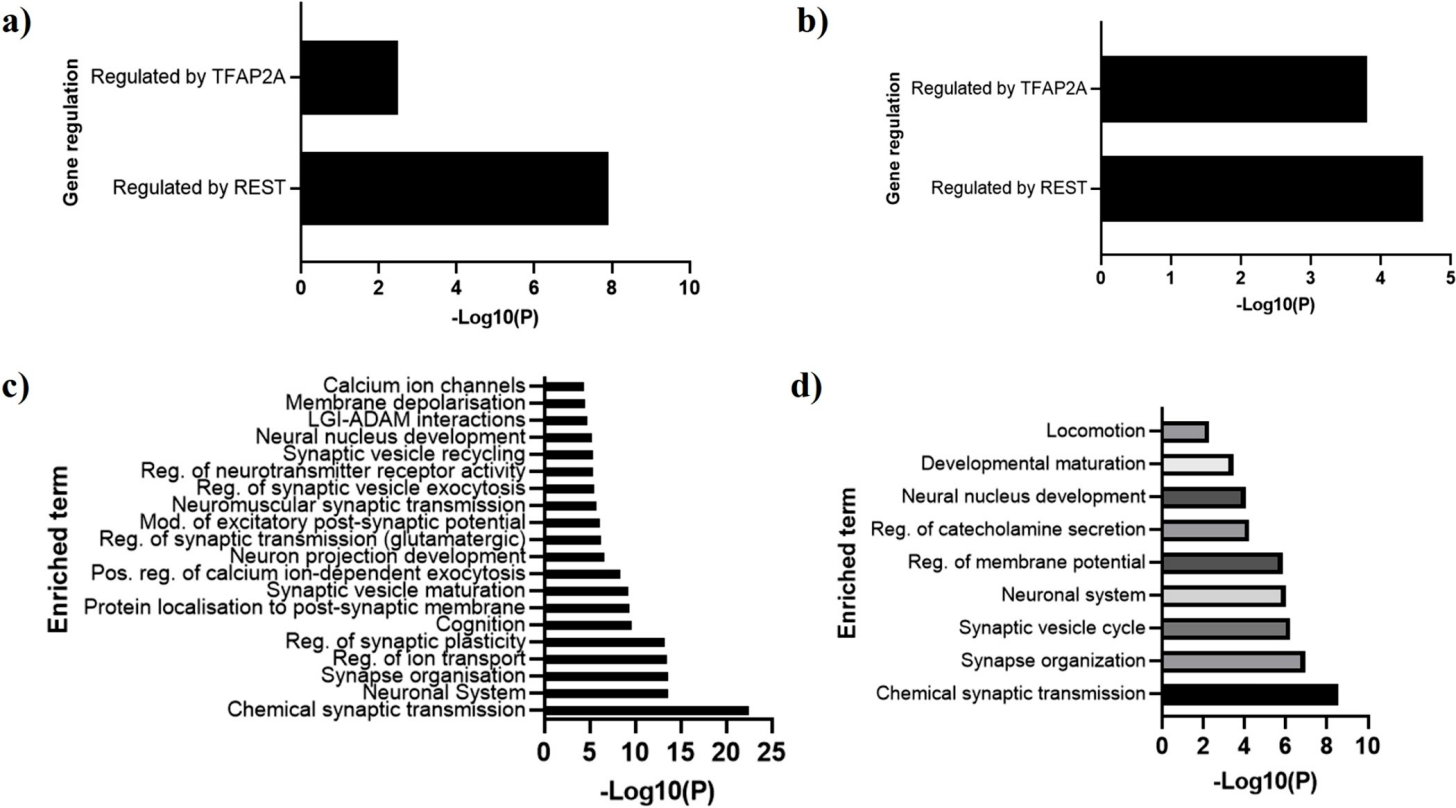
Gene ontology analysis of *HAR1A* and *HAR1B* coexpressed genes in lower grade gliomas. Analysis of **a)** *HAR1A* upstream regulators, **b)** *HAR1B* upstream regulators, **c)** *HAR1A* downstream pathways (enriched terms), **d)** *HAR1B* downstream pathways. Analysis conducted on Metascape. -Log10(P) is p-value in log base 10.

After showing that *HAR1A* and *HAR1B* have positive prognostic roles in lower grade gliomas, we determined possible downstream pathways and upstream regulators of the lncRNAs, using gene ontology (Fig 3). The genes most significantly coexpressed with *HAR1A* and *HAR1B* in lower grade gliomas were analyzed in the publicly available ontology resource Metascape. As expected from the previous analysis, we identified REST as the most significant regulator of both lncRNAs (Fig 3A and 3B). Further to this analysis, we also determined the most likely downstream pathways of *HAR1A* and *HAR1B* (Fig 3C and 3D): chemical synaptic transmission and other synapse-related pathways.

### Downstream pathways associated with REST

**Fig 4:**
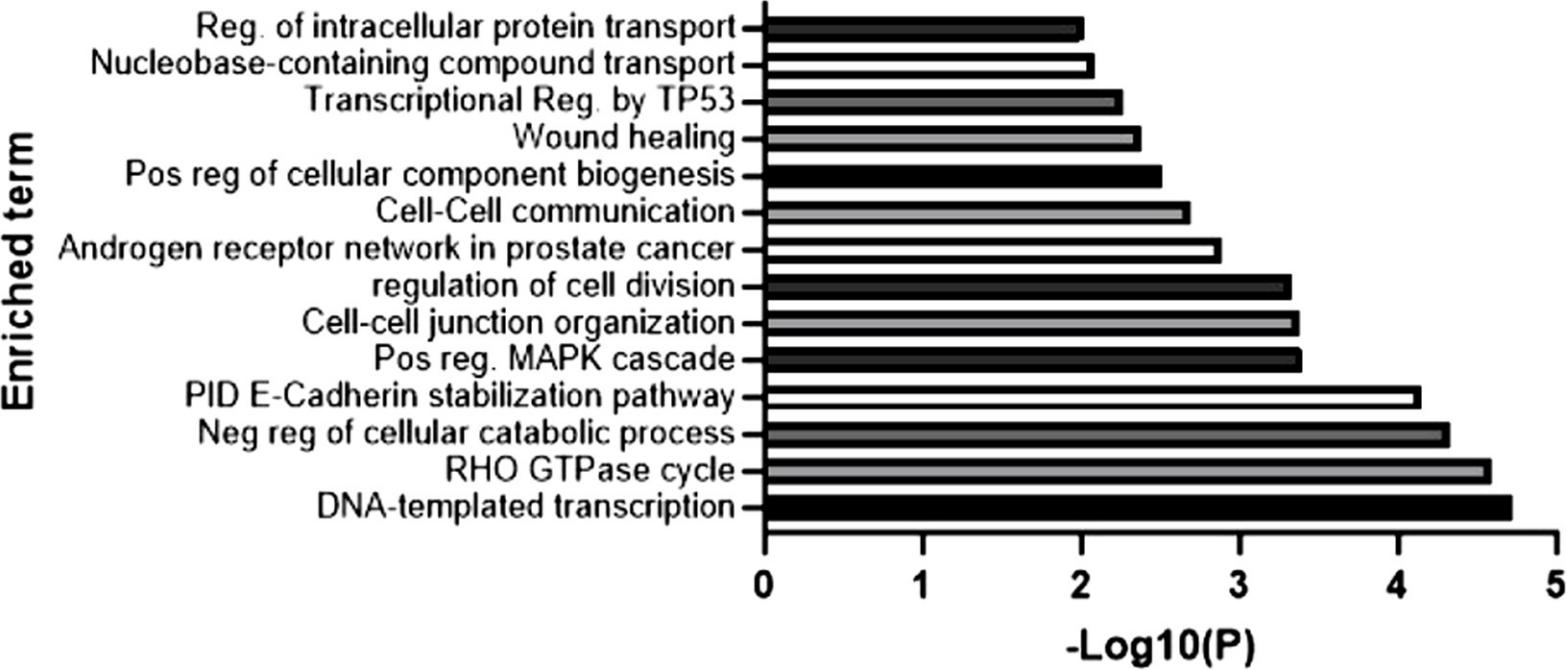
Downstream pathways associated with REST. Gene ontology analysis with REST co-expressed genes, associating downstream pathways (enriched terms). Analysis conducted on Metascape. -Log10(P) is p-value in log base 10(P) is *p*-value in log base 10.

Using the same dataset for *REST*-correlated genes, we found that *REST* expression is associated with several cancer-relevant pathways (Fig 4), including GTPase activity, TP53 regulation, MAPK cascade, cell division, and wound healing.

### Expression of REST, HAR1A, and HAR1B in glioma cells

**Fig 5:**
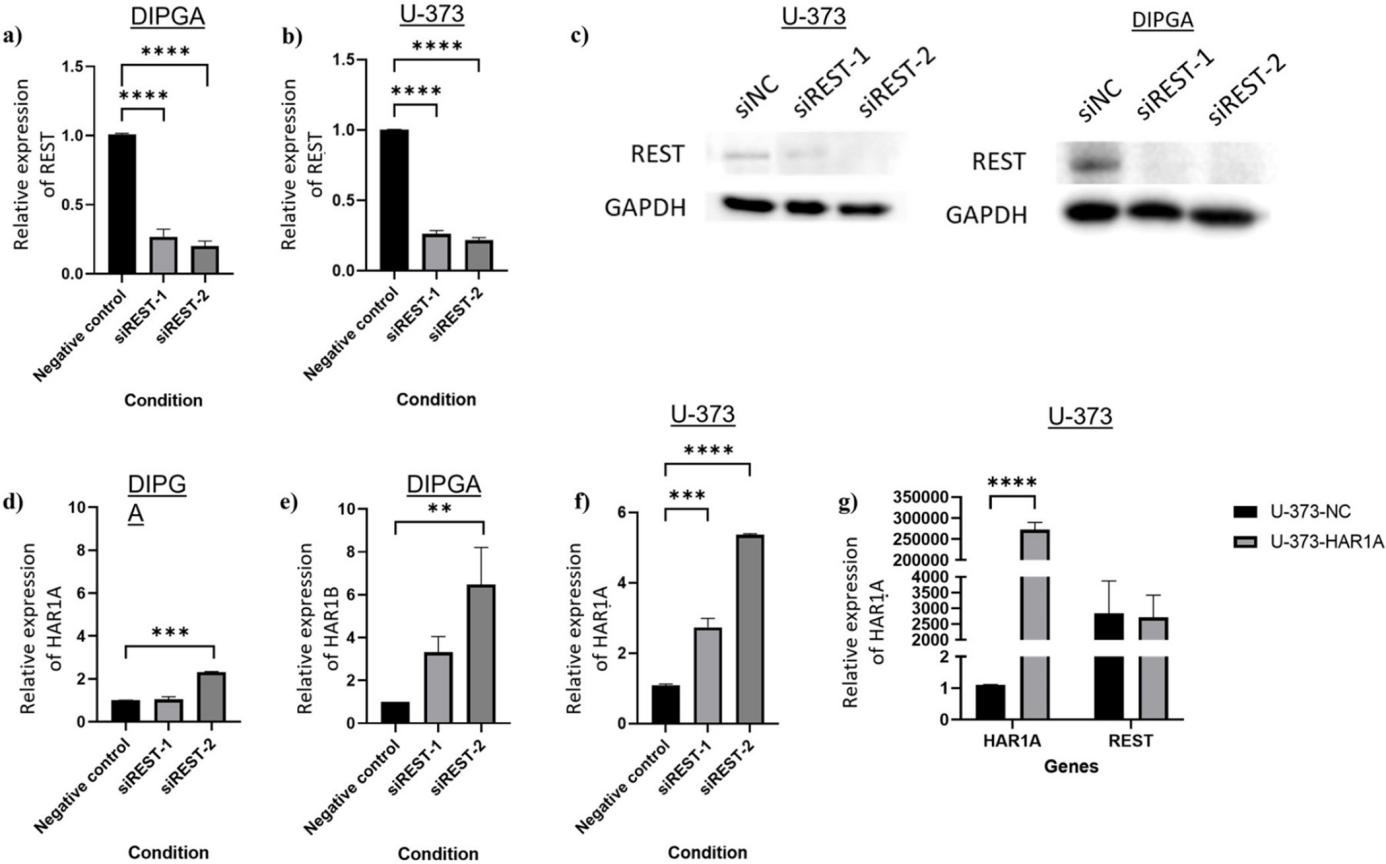
The expression of *HAR1A* and *HAR1B* in U-373 and DIPGA cell cultures. *REST* silencing using two independent siRNAs in **a)** DIPGA and **b)** U-373. **c)** Western blot of REST levels when gene silenced in U-373 and DIPGA cells. **d)** *HAR1A* expression upon *REST* silencing in DIPGA, **e)** *HAR1B* expression upon *REST* silencing in DIPGA, **f)** *HAR1A* expression upon *REST* silencing in U-373. **g)** *HAR1A* and *REST* expression upon *HAR1A* overexpression and control in U-373. In a,b,d-g) bars represent the mean values of 3 independent experiments with error bars denoting SEM. *GAPDH* was used as the housekeeping gene. One-way ANOVA with Dunnett’s multiple comparison tests used to compare the siRNAs silenced or *HAR1A* overexpressing cells to the negative control. ** p<0.01, ***p<0.005, ****p<0.001. N = 3.

Following the bioinformatic analysis, we observed low expression of *HAR1A* and *HAR1B* in U-373 (GBM) and DIPGA (DMG) cell cultures (Ct>35). To understand whether these expression levels could be functionally relevant, we compared *HAR1A* expression in glioma *vs* other cell types, using the human Expression Atlas [13]. Among 16 organs, the brain and the prostate showed the highest levels of *HAR1A* (S2A Fig). We therefore measured *HAR1A* levels in prostate cancer cells (LNCaP), finding that *HAR1A* has a higher expression in the prostate cancer cells, compared to GBM and DMG cells (S2B Fig). To the contrary, REST expression was highly detectable in both glioma cells, as shown in previous studies [14]. Therefore, we silenced *REST* using siRNAs, as shown previously [10] which resulted in increased expression of *HAR1A* and *HAR1B* in both DIPGA and U-373 cell lines (Fig 5A, 5B and 5D–5G). Moreover, we measured protein levels by western blot, confirming that REST protein expression was reduced in both cell lines (Fig 5C). For the siRNA that we used, siREST-2 significantly increased *HAR1A* and *HAR1B* in DIPGA cells, likely because this siRNA had the largest silencing effect on *REST* (Fig 5D and 5E). Next, in U-373 cells we found that *REST* silencing significantly increased the expression of *HAR1A* (Fig 5F), however, *HAR1B* was not expressed in this cell line.

In parallel with the *REST* silencing model, we also stably overexpressed *HAR1A* in the U-373 cell line (Fig 5G) and observed that the expression of REST did not change between the control and *HAR1A* overexpression.

**Fig 6:**
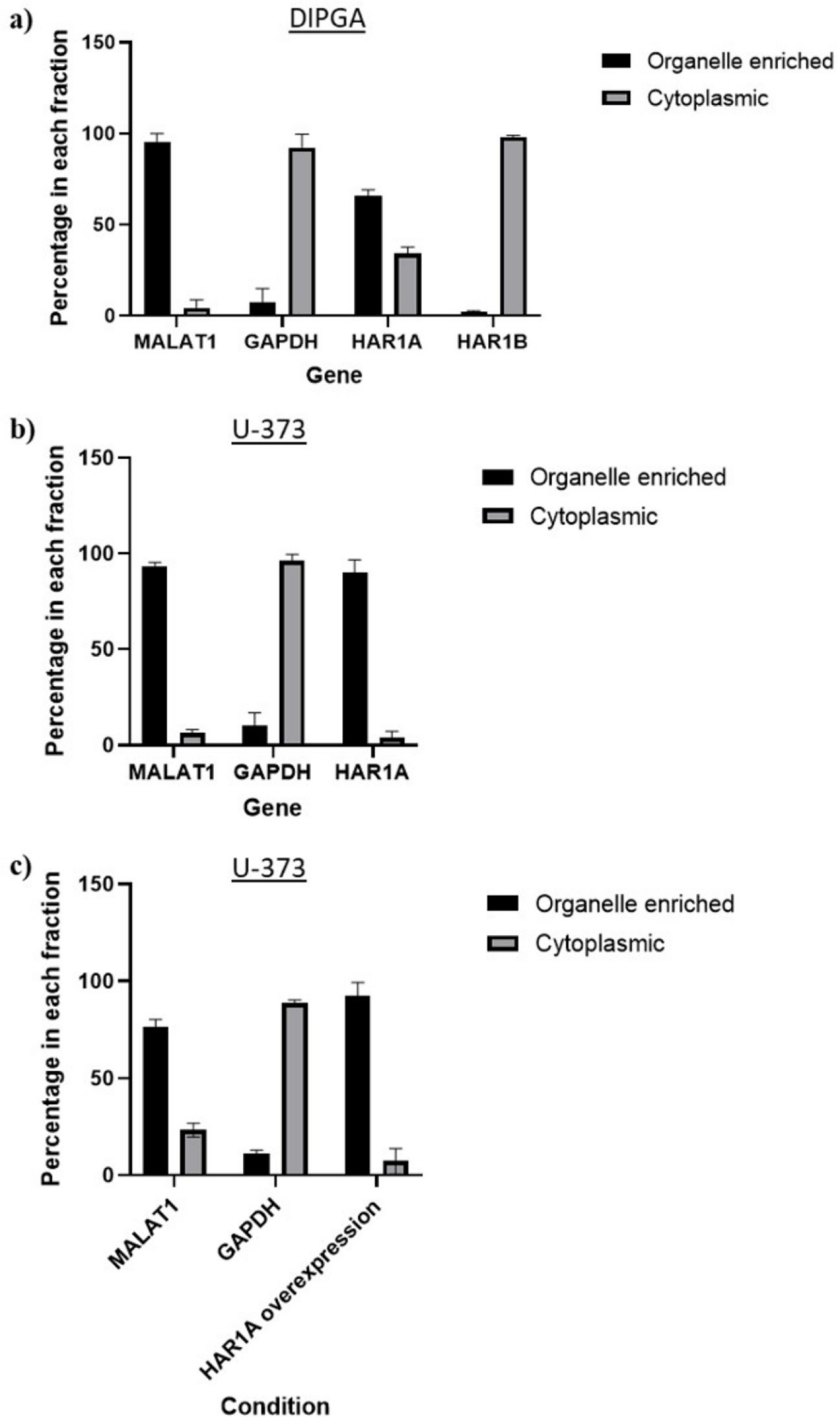
Subcellular localization of *HAR1A* and *HAR1B* transcripts. Expression of **a)** *HAR1A* and *HAR1B* in DIPGA cells, **b)** *HAR1A* in U-373 cells, **c)** *HAR1A* in *HAR1A* overexpressed U-373 cells. *MALAT1* is nuclear control, and *GAPDH* is cytoplasmic control. *HPRT1* was used as the housekeeping gene. N = 3.

After determining the expression of *HAR1A* and *HAR1B* in the glioma cell lines, we identified their subcellular localization (Fig 6). The lncRNA expression was analyzed by cell fractionation and RT-qPCR in DIPGA (Fig 6A), U-373 (Fig 6B) and *HAR1A-*overexpressed U-373 (Fig 6C) cell lines. We found *HAR1A* to be primarily localized in the organelle enriched component in all tested cell lines (which is mostly nuclear, as independently validated in S3 Fig), whereas *HAR1B* to be primarily cytoplasmic in DIPGA cells. Using specific markers of nuclear and cytoplasmic fractions, we independently validated our cell fractionation results. Localization of *HAR1B* was not determined in cells U-373 (Fig 6B) and *HAR1A-*overexpressed U-373, as *HAR1B* was not expressed in these cell lines.

### Effects of REST and HAR1A on glioma cell proliferation

**Fig 7:**
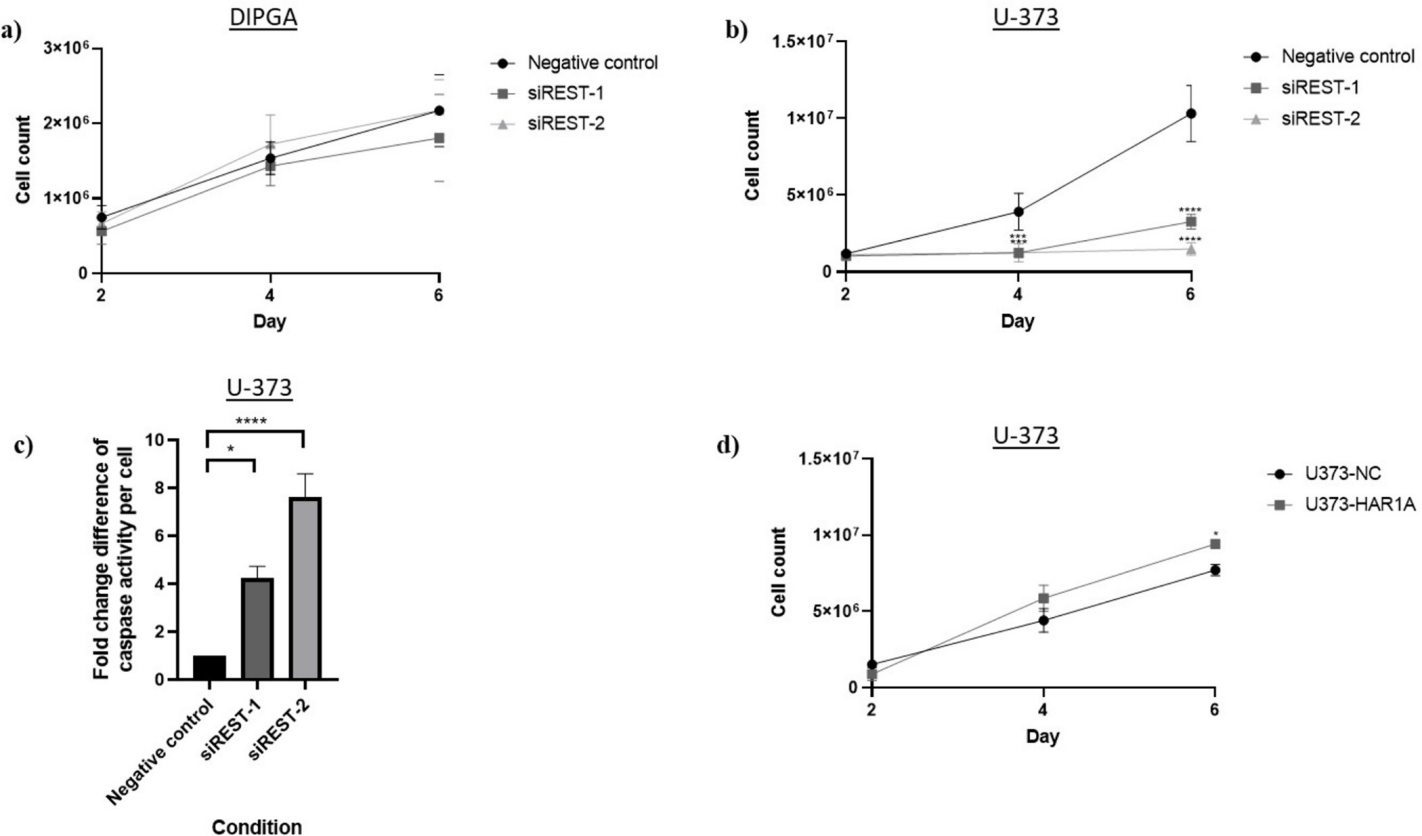
Effects of HAR1 and REST modulation on apoptosis and proliferation. **a)** Cell count assay with *REST*-silencing in DIPGA cells. **b)** Cell count assay with *REST*-silencing in U-373 cells, **c)** apoptosis assay in U-373 cells, d) cell count assay in U-373 *HAR1A*_overexpression cell line. Two-way ANOVA with Dunnett’s multiple comparison test used to compare the siRNAs silenced or *HAR1A* overexpressed cells to the negative control in the cell count assays. One-way ANOVA with Dunnett’s multiple comparison test used to compare the siRNAs silenced cells to the negative control in the apoptosis assay. * p<0.05, ** p<0.01, ***p<0.005, ****p<0.001. N = 3. Two-way ANOVA with post-hoc multiple comparison test for the HAR1A OE experiment in d).

Having identified the subcellular localization of the lncRNAs in the cell, we then investigated whether *REST* silencing and *HAR1A* overexpression affect the cell proliferation and apoptosis of cancer cells (Fig 7). *REST* was silenced with siRNAs in DIPGA cells and U-373 cells, then cell counts were taken every 2 days over 6 days. There was no significant difference between the control and *REST*-silenced cells for DIPGA cells (Fig 7A). For U-373 cells, we found *REST* silencing significantly reduced cell proliferation, compared to the control (Fig 7B; 68–86% decrease *vs* siRNA control). A caspase 3/7 apoptosis assay was performed on these cells on day 4 of *REST*-silencing (Fig 7X). This showed that *REST* silencing resulted in an increase in caspase activity in U-373 cells. To identify whether this difference in U-373 cell proliferation and survival was mediated by the REST-dependent increase in *HAR1A* expression, we repeated the cell count using a *HAR1A* overexpression and negative control models (Fig 7D). Here, we found no difference between the control vector and *HAR1A* overexpressing cells in 2 out of 3 timepoints (2 and 4 days). *HAR1A* overexpression seemed to induce a slight (albeit significant) increase in cell proliferation after 6 days (22% increase *vs* control vector).

Taken together, these data and the results of the previous section show that REST inhibits the expression of *HAR1A* in glioma cells, and that REST appears to be essential for the proliferation of GBM cells. However, *HAR1A* modulation alone does not seem to have an inhibitory effect on the proliferation of GBM cells.

### Effects of HAR1A on glioma cell migration, nuclear structure, and differentiation

**Fig 8:**
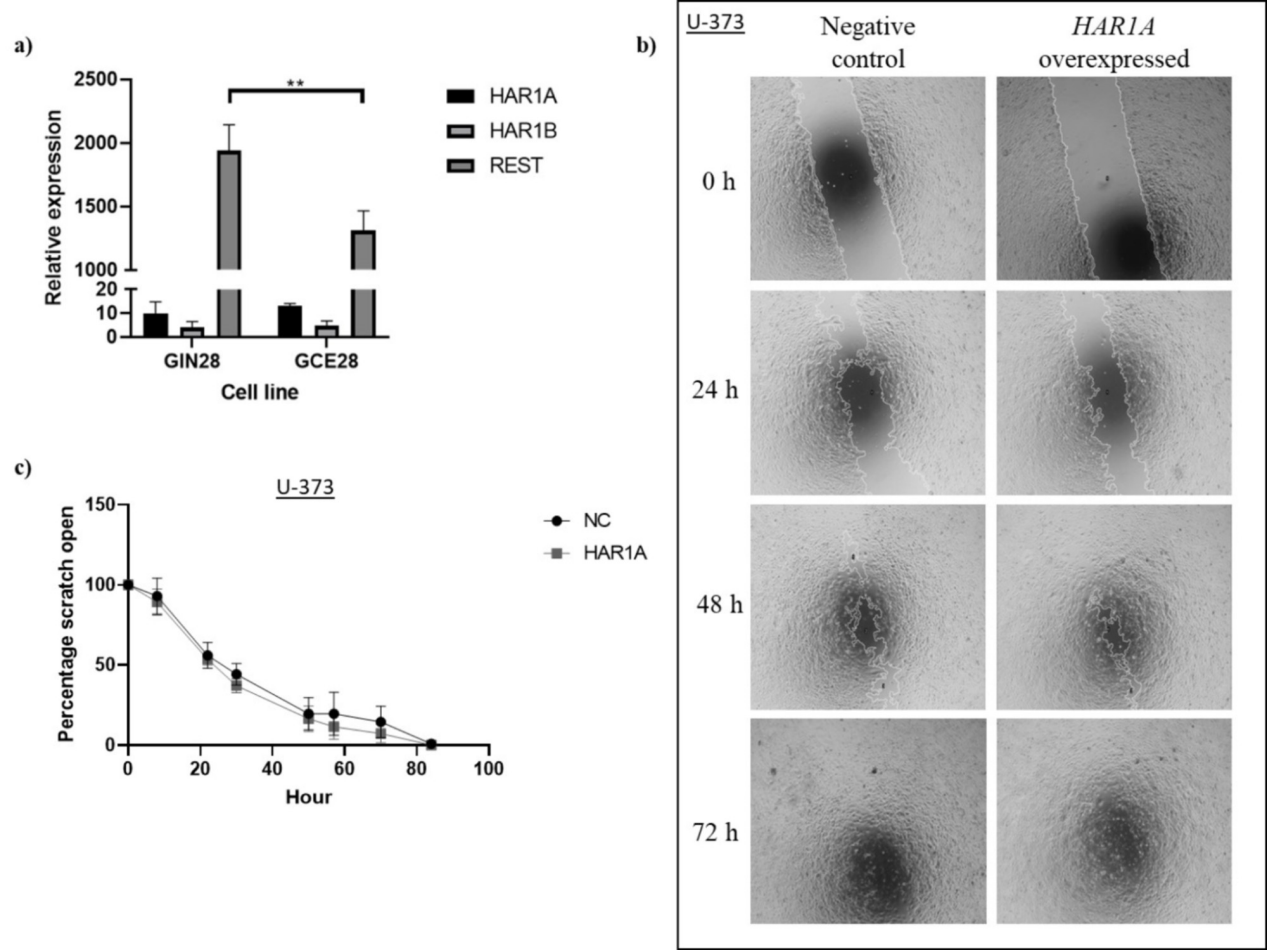
*HAR1A* and glioblastoma cell migration. **a)** *HAR1A*, *HAR1B* and *REST* expression in GIN28 (invasive margin) and GCE28 (tumour core), data obtained with RT-qPCR; **b)** Images of the wound closing over 72 hours, **c)** percentage of the scratch is open for *HAR1A* U-373 control and overexpression. *GAPDH* was used as the housekeeping gene. For **a)**, two-way ANOVA with Šídák’s multiple comparison test was used to compare the gene expression between the two cell lines. **p<0.01. N = 3.

**Fig 9:**
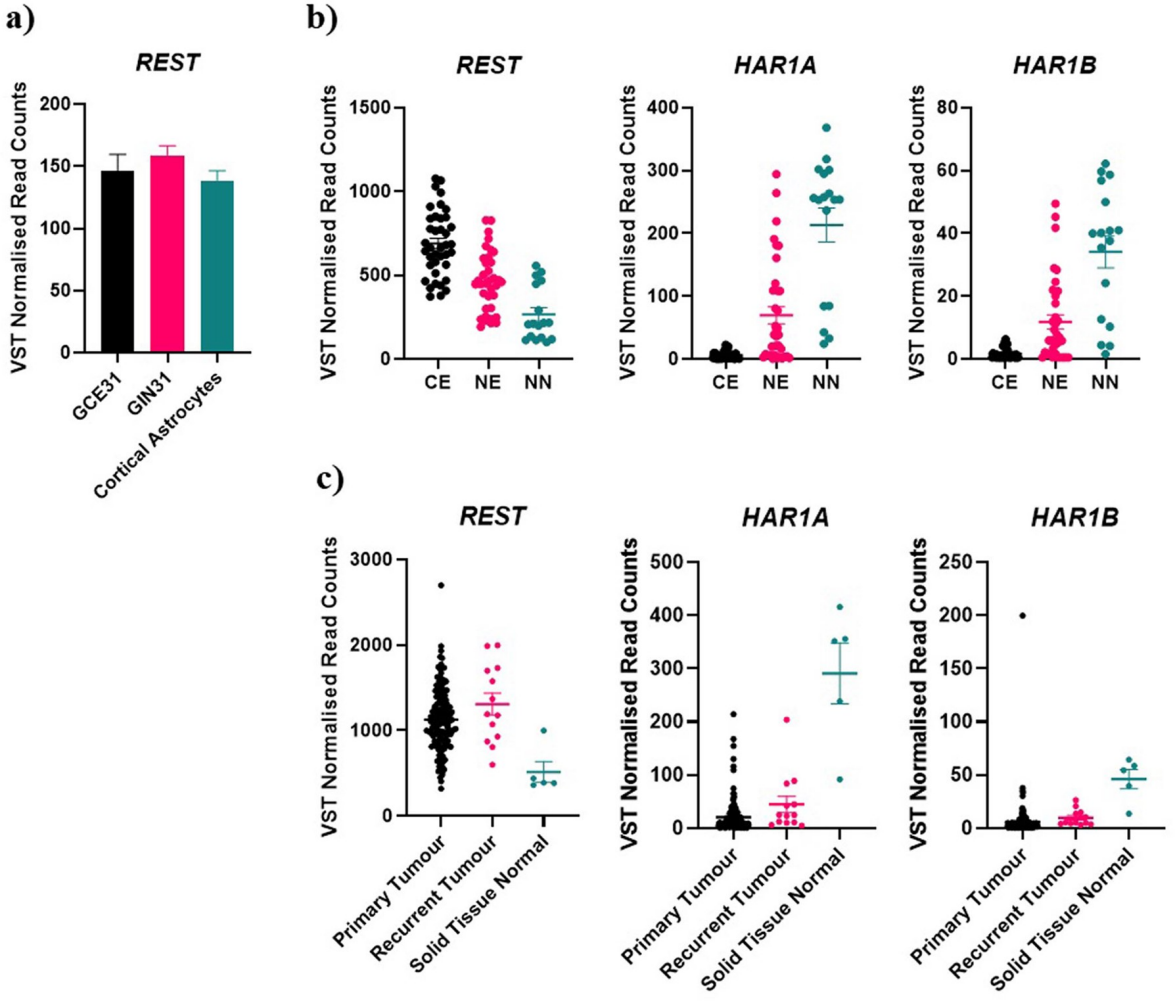
Validation of gene expression in external datasets. **a)** VST normalised gene counts were not differentially expressed between GIN31 and GCE31 (n = 3) (DESeq2 DEA adjusted p value > 0.05), mean + SEM. **b)** VST normalised gene read counts from MRI localised tissue samples from the tumour core (CE, contrast enhancing, n = 39) tumour margin (NE, non-enhancing, n = 36), and non-neoplastic (NN, from access biopsy from patients undergoing ventriculoperitoneal shunt placement, n = 17) controls. DESeq2 DEA showed that *REST* (Log2 Fold change -0.58, adjusted *p* value 1.708477e-05), *HAR1A* (Log2 Fold Change 3.74, adjusted *p* value 2.310446e-17), and *HAR1B* (Log2 Fold Change 3.24, adjusted *p* value 1.059286e-11) were differentially expressed between NE and CE tumour regions. **c)** VST normalised gene read counts from unmatched Primary (n = 158) and Recurrent (n = 13) tumour samples and non-tumour brain tissue (n = 5) controls from the TCGA-GBM cohort. DESeq2 DEA showed no difference in *REST*, *HAR1A*, and *HAR1B* expression between recurrent and primary tumours (adjusted *p* value > 0.05).

In addition to its role in cell proliferation, REST has been previously shown to be a key driver of glioma cell migration [14]. For this reason, we analyzed the expression of *REST* and *HAR1A* in primary GBM cells derived from the tumour core and from the invasive margin of the same patient (patient 28) [15]. Our data showed a significant up-regulation of *REST*, but not *HAR1A* in the invasive margin, relative to the tumour core (Fig 8A). To validate against paired gene expression data of tumour core and invasive margin cell lines from an independent patient (patient 31) we analysed untreated controls from published RNA-seq data from our previous study of GIN31 and GCE31 response to electric field stimulation [16]. In contrast, *REST* was not significantly upregulated in GIN31 compared to GCE31 (Fig 9A). To further investigate the clinical relevance of *REST* and *HAR1A/HAR1B* expression, we compared the expression of these genes in a publicly available dataset of glioblastoma samples taken from MRI contrast enhancing (CE) tumour core and non-enhancing (NE) tumour periphery regions [17]. *REST*, *HAR1A*, and *HAR1B* were all differentially expressed between the tumour core and periphery samples (Fig 9B). However, as the non-enhancing region contains both invasive glioblastoma cells and surrounding parenchyma and this change is consistent with comparison to healthy brain controls, we are unable to comment on expression of *REST* in invasive glioblastoma relative to core in these samples. Finally, as glioblastoma invasive cells are known to drive tumour recurrence, we analysed expression of these genes in primary and recurrent glioblastoma samples from TCGA. We identified 595 differentially expressed genes (padj < 0.05 and absolute log2 fold change > 1), however there was no significant difference in *REST*, *HAR1A*, or *HAR1B* gene expression between these unmatched primary and recurrent samples (Fig 9C). Conversely, relative to normal solid tissue samples, *REST* was upregulated in primary and recurrent tumour samples and *HAR1A* / *HAR1B* were downregulated in both primary and recurrent tumours (Fig 9C).

In keeping with this observation, we did not find any changes in cell migration between the *HAR1A* overexpression model and control in U-373 cells, using a scratch assay (Fig 8B, 8C).

**Fig 10:**
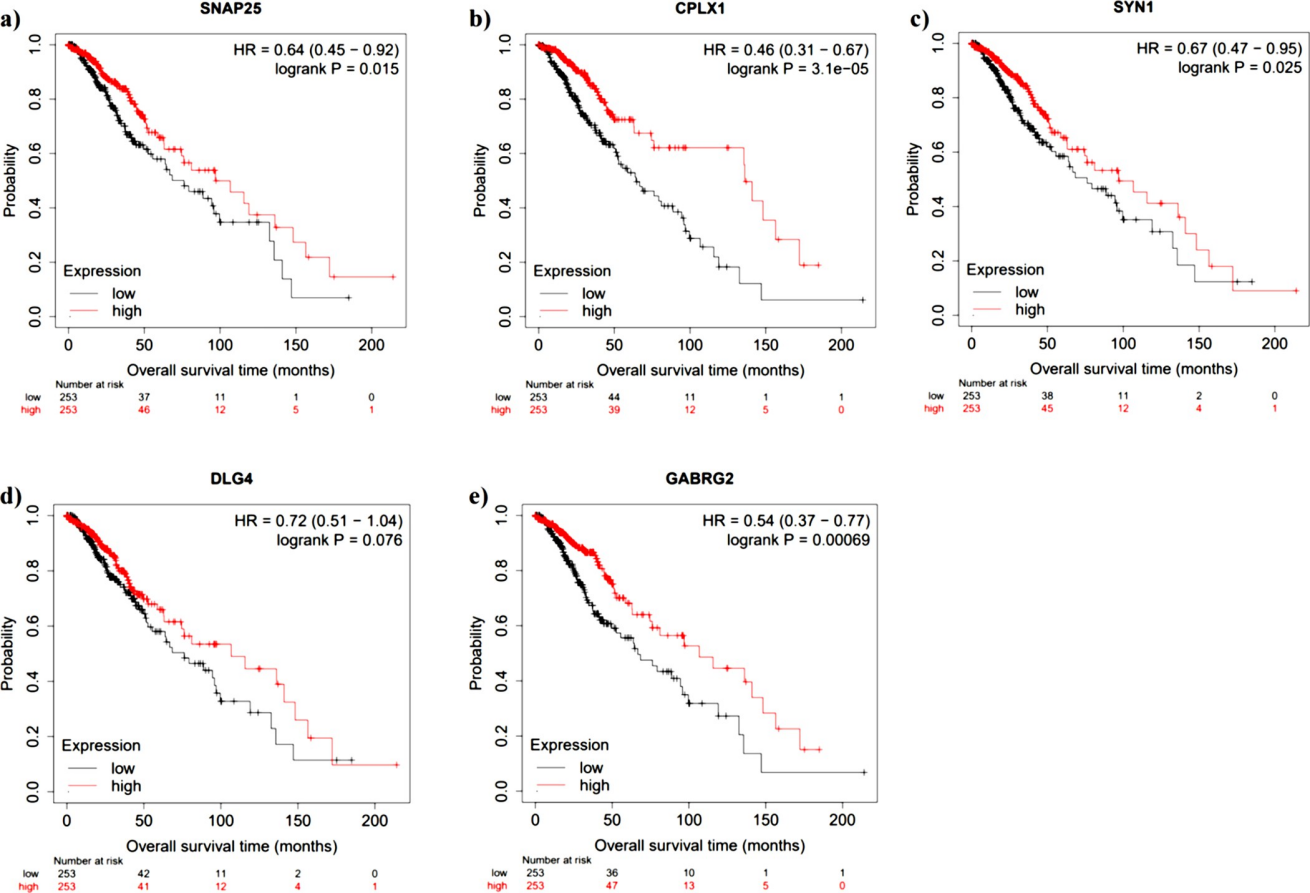
The overall patient survival of glioma patients depending on synaptic gene expression. Kaplan-Meier plots from KMP-Plotter show high expression (red) or low expression (grey), relative to the median. **a)** High expression of *SNAP25* results in worse patient survival. **b)** High expression of *CPLX1* results in worse patient survival. **c)** High expression of *SYN1* results in worse patient survival. **d)** High expression of *DLG4* results in worse patient survival. **e)** High expression of *GABRG2* results in worse patient survival.

Having not yet found sufficient evidence of a functional role of *HAR1A*, we next investigated the correlations between the expression of *HAR1A* and synaptic genes. This is based upon the previous gene ontology results indicating that *HAR1A* may have a role in synaptic transmission (Fig 3C). Based on our pathway analysis, 15 synaptic genes were significantly associated with *HAR1A* expression. We investigated the prognostic role of these genes through the Kaplan Meier Plotter dataset (Fig 10). Five synaptic genes were selected based on their prognosis status in gliomas (higher expression = better prognosis): *SNAP25* (encoding Synapsis Associated Protein 25), *CPLX1* (encoding Complexin 1), *SYN1*, *DLG4* (encoding Postsynaptic Density Protein 95), and *GABRG2* (encoding Gamma-Aminobutyric Acid Type A Receptor Subunit Gamma2).

**Fig 11:**
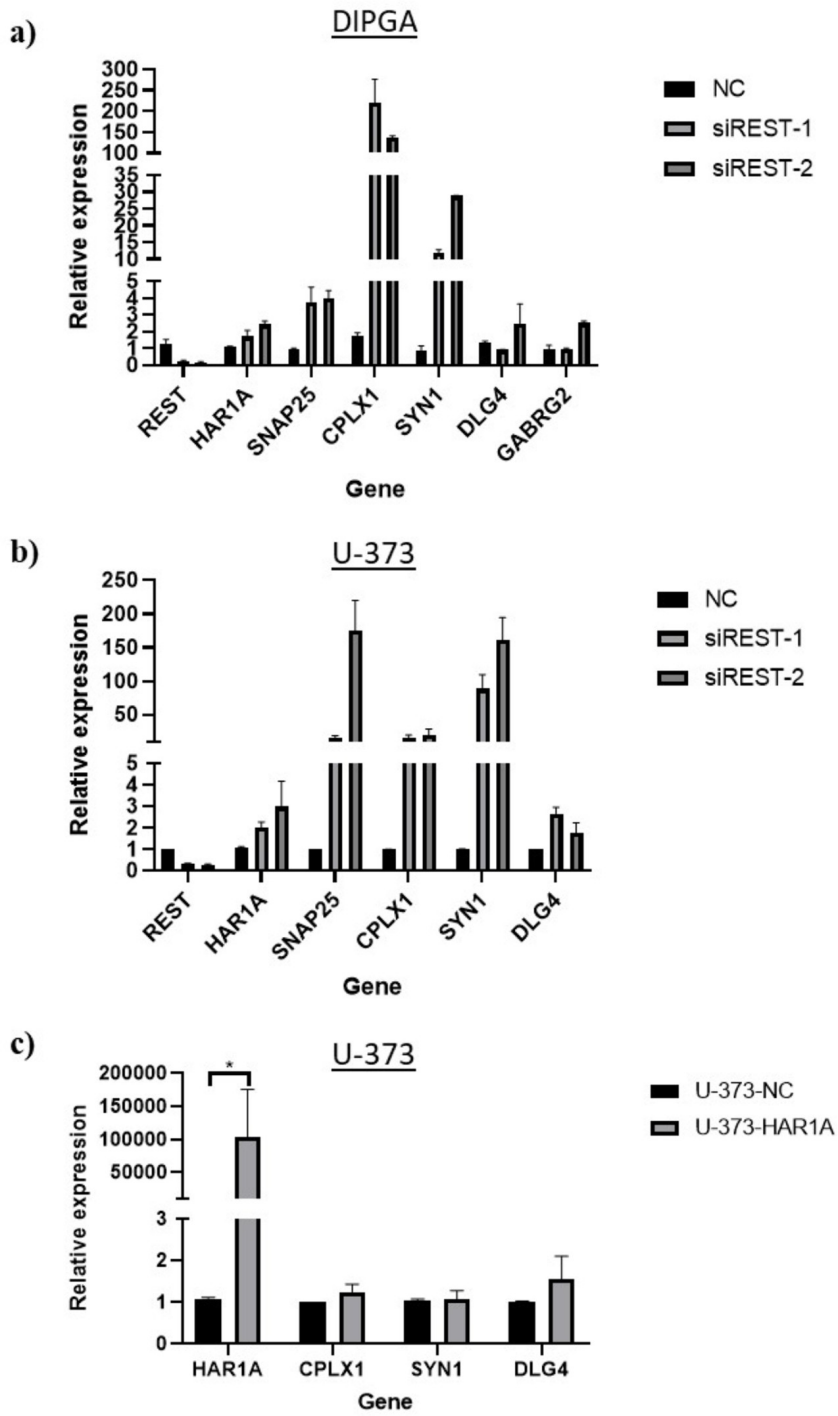
Synaptic gene correlation with *HAR1A*. **a)** Expression of synaptic genes in *REST*-silenced DIPGA cells, **b)** Expression of synaptic genes in *REST*-silenced U-373 cells, **c)** expression of synaptic genes in *HAR1A*-overexpressed U-373 cells. * p<0.05. N = 3.

The expression of these genes was measured by RT-qPCR in siREST-silenced DIPGA and U-373 cells and *HAR1A*-overexpressed cells (Fig 11). Our results showed that *REST* silencing caused the overexpression of CPLX1, SYN1 and SNAP25 in DIPGA (Fig 11A), and all synaptic genes in U-373 (Fig 11B). However, we did not find any effects of *HAR1A* overexpression on the expression of the same genes (Fig 11B).

Overall, our results show that REST exerts pleiotropic pro-oncogenic functions in gliomas, as shown by its pro-survival and anti-apoptotic effects, and by its higher expression in invasive glioma cells. As expected, REST suppresses the expression of *HAR1A* in both pediatric and adult glioma cells. However, none of the oncogenic roles of REST seem to be mediated by *HAR1A*.

## Discussion

In this study, we have shown that *HAR1A* is negatively regulated by REST in glioma cells, and that the expression of these two molecules has an opposite prognostic significance in glioma patients. Our results are in line with previous publications [18] but expand these findings on two key points: (1) we show that the prognostic role of these molecules is more relevant in lower grade gliomas, compared to GBM. This may be due to the extremely short survival time of GBM patients, which makes prognostic stratification more challenging; (2) we show that REST exerts its oncogenic function through different mechanisms that include enhancing GBM cell proliferation and survival, as well as reducing the expression of neural differentiation genes (but largely/mostly not via *HAR1A* repression). Loss of neural differentiation is a key step in gliomagenesis [19]. REST is a known negative regulator of neural differentiation, with repressive effects on the expression of SNAP25 [20] and SYN1 [21]. However, our results show that REST also inhibits the expression of CPLX1 and DLG4. CPLX1 is a protein required for the calcium-dependent exocytosis of synaptic vesicles [22]. DLG4 is a post-synaptic scaffold protein [23], which has been implicated in glioma pathogenesis by previous bioinformatic analysis [24]. To the best of our knowledge, the function of the CPLX1 protein has never been described in gliomas. Here we show that the *CPLX1* gene is suppressed by REST and that higher CPLX1 expression predicts better prognosis in gliomas. These findings warrant further exploration of the REST-CPLX1 axis.

To broaden the significance of our findings, we have employed two glioma cell lines with different characteristics: one deriving from an adult GBM and the other from a pediatric glioma. The results obtained with the two cell lines are only partially overlapping, probably because of the heterogeneous nature of gliomas. Whilst the regulation of *HAR1A* by REST seems to be a cell type-independent mechanism, the effects of *REST* silencing on proliferation were much more marked in GBM than in DMG cells. Furthermore, we confirmed that *HAR1A* and *HAR1B* expression is comparable intra-tumour cell lines derived from both the GBM core and clinically relevant invasive margin, the latter region reflective of residual disease post-surgery.

Due to the strong negative correlation between REST and *HAR1A*, we have hypothesized that the oncogenic activity of REST in glioblastoma was at least in part mediated by *HAR1A* silencing. For this reason, we have overexpressed *HAR1A*, and confirmed that this transcript is probably localized in the nucleus, where it has been shown to have onco-suppressive functions [18]. Our overexpression studies did not corroborate a tumour suppressive role for *HAR1A* in glioma. The overexpression of this lncRNA had no inhibitory effect on cell proliferation and migration. If anything, we found that the overexpression of *HAR1A* could slightly increase the proliferation of GBM cells. This appears to be in contrast with our prognostic findings and with other studies describing a tumour suppressive role for *HAR1A*. However, the magnitude of *HAR1A* effect on proliferation is modest (22% increase; compared to a 68–86% reduction in proliferation upon REST silencing). Since REST silencing induces both cell death and *HAR1A* over-expression, it is likely that the slight pro-survival effects associated with *HAR1A* upregulation are counterbalanced by the inactivation of several other REST-dependent pro-survival pathways. In keeping with this hypothesis, a recent study showed that REST silencing down-regulates several pathways involved in glioma cell mitosis and cell proliferation [25]. This hypothesis is corroborated by our GO Pathway analysis (Fig 4), showing that *REST* expression is correlated with key oncogenic pathways (*e*.*g*., MAP kinase, cell division, wound healing). Synaptic reprogramming is emerging as a key driver of oncogenesis in gliomas [26]. We were therefore intrigued to identify a strong bioinformatic signal linking synaptogenesis with *HAR1A* expression. However, our RT-qPCR experiments (Fig 11) shows that this lncRNA does not regulate the expression of synaptogenesis genes in GBM. Nuclear structure and nuclear/cytoplasm ratio alterations are another hallmark of cancerogenesis [27], which may be controlled by lncRNAs. Hence it may be conceivable that *HAR1A* affects this aspect of carcinogenesis. Notably, HAR1A-downstream pathways in gliomas have not been explored so far. This potential function should be therefore investigated by future studies.

Our study provides new insights into the REST-*HAR1A* relationship in gliomas; however, we recognize that our investigation has some limitations. First, we studied the role of *HAR1A* in a limited collection of cell types (glioma and glioblastoma). *HAR1A* is also expressed in neurons [7] and in other cell types, including the prostate (S2 Fig), lung epithelium [9] and peripheral blood [28]. It is therefore conceivable that *HAR1A* could have a functional role in these cell types, which are not the object of our investigation. In addition, our overexpression system induced very high levels of *HAR1A* expression, which are likely non-physiological. The lack of measurable phenotypes in these conditions further suggests a minor role of this lncRNA in GBM cells. Our GO term analyses (Figs 3 and 4) are based on co-expression between *REST*/*HAR1* and protein coding genes. These correlations are not a definitive mechanistical explanation of the pathways activated by these genes, but they have provided valuable insights into REST-dependent regulatory pathways, some of which we have experimentally confirmed (Fig 11). Finally, we would like to clarify that our overexpression experiments rule out a *trans* role for this lncRNA, but do not completely exclude a *locus*-specific function (*cis*) for this transcript. Also, given the known role of REST in glial migration [29], and its up-regulation in invasive glioma cells, it would have been interesting to test whether silencing this gene affected the migratory properties of invasive glioma cells. However, since REST silencing caused extensive cell death in a relatively short timeframe, the migration experiment could not be performed.

## Conclusions

We show that REST can impact the prognosis and progression of gliomas via different mechanisms, including enhanced cell proliferation, repression of neural-differentiation genes and potentially increased migration. Our results seem to exclude an *in vitro* function for *HAR1A* in gliomas. This does not exclude an *in vivo* function for this lncRNA, perhaps mediated by the interaction between the microenvironment and malignant cells. This function could be studied in the future using animal models or 3D co-cultures. Considering the ongoing development of REST small molecule inhibitors for glioma treatment [30], our results have therapeutic relevance. We show that some REST-downstream effects (e.g. pro-survival signals, silencing of neural differentiation genes) are more therapeutically relevant than others (e.g. *HAR1A* silencing). This evidence may inform the selection of more promising inhibitors and the identification of biomarkers of efficacy in pre-clinical and clinical studies.

## Supporting information

S1 Fig Clinical significance of *HAR1A*, *HAR1B* and *REST* in paediatric gliomas. (A-C): Linear regressions in paediatric gliomas of A) *HAR1A* and *REST*, B) *HAR1B* and *REST*, C) *HAR1B* and *HAR1A*. (D-F): Overall survival of paediatric glioma patients according to the expression of D) *REST*, E) *HAR1A*, F) *HAR1B*. Data from the Paediatric Cbioportal (PTBA-provisional, 2182 samples). Gene expression (mRNA) is measured as Z scores, log2 scale.
(TIF)

S2 Fig *HAR1A* expression in a panel of organs and cell lines. (A) Expression plot of *HAR1A* from the Illumina Body Map (https://www.ebi.ac.uk/). (B) RT-qPCR (Ct values) of HAR1A expression in LNCaP (prostate), U-373 (brain) and DIPGA (brain) cells. GAPDH was used as reference.
(TIF)

S3 Fig Validation of nuclear and cytoplasmic fractionation. Immunoblotting analysis of preparation of separate nuclear and cytoplasmic lysates from cancer cells trough PARIS Kit protocol (Invitrogen, Cat #AM1921). Cytoplasmic beta-Tubulin (Cell Signaling Cat #2128S) and GAPDH (Cell Signaling Cat #5174T) proteins were adopted to evaluate potential cytosolic contaminations in the nuclear fraction. Lysosomal LAMP1 (Cell Signaling Cat # 9091) protein was used to evaluate potential contamination of organelles in the nuclear fraction. Histone H3 (Invitrogen Cat # 710282) protein was used to confirm that the fraction separated from cytosolic fraction was nuclear.
(TIF)

S1 Raw images Raw blot images. This Supporting Information file has raw blots showing expression of REST and GAPDH in U-373 cells from Fig 5c; REST and GAPDH in DIPGA cells from Fig 5c; as well raw blots showing expression of LAMP1, beta-tubulin, GAPDH, histone H3 from S3 Fig.
(PDF)
