# Supplementary figures and images for: REST-dependent glioma progression occurs independently of the repression of the long non-coding RNA HAR1A
Source: PLoS One. 2024 Nov 27;19(11):e0312237. doi: 10.1371/journal.pone.0312237 (PMC11602025; doi:10.1371/journal.pone.0312237)

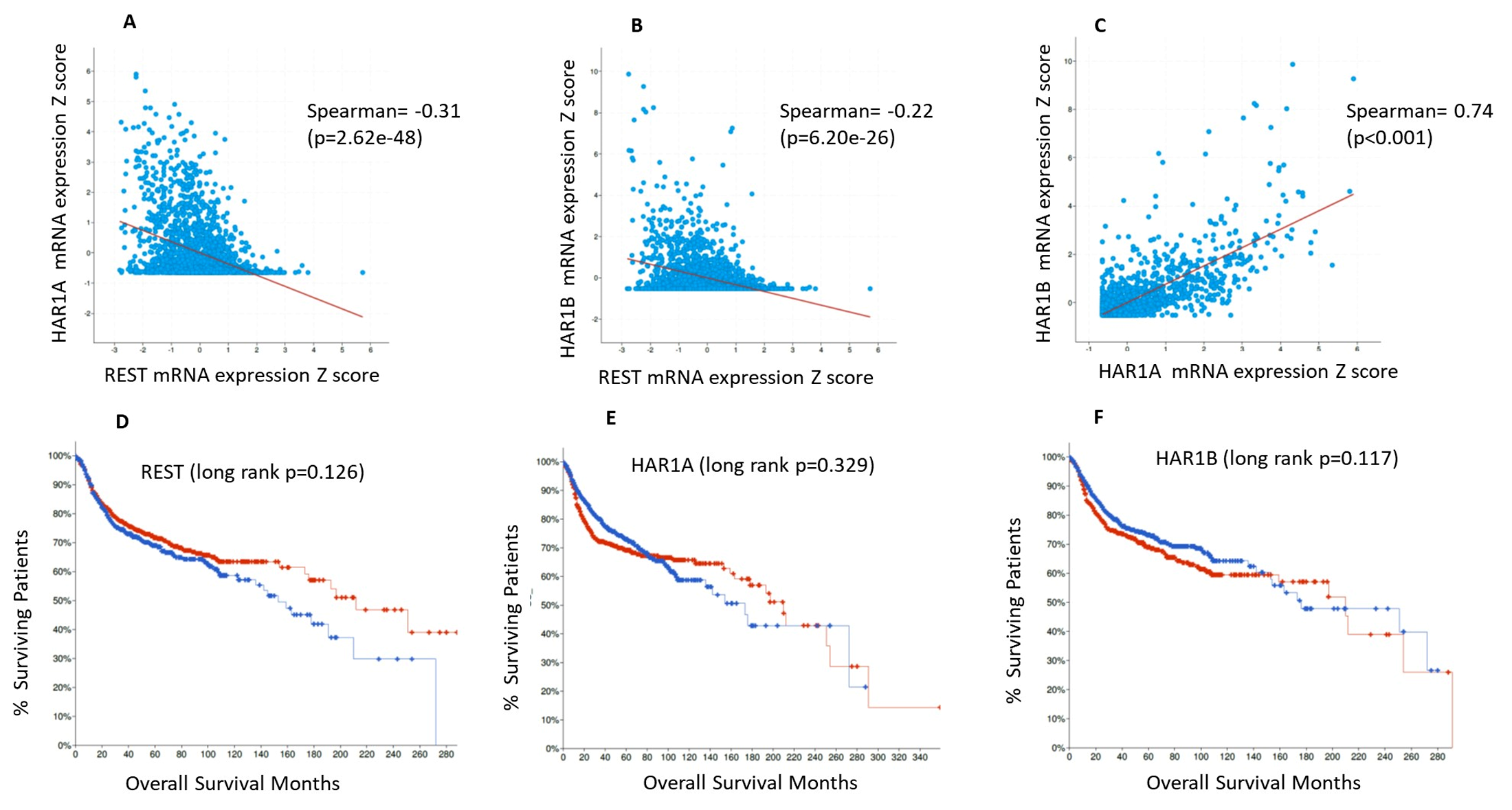

Supplement: S1 Fig — (A-C): Linear regressions in paediatric gliomas of A) HAR1A and REST, B) HAR1B and REST, C) HAR1B and HAR1A. (D-F): Overall survival of paediatric glioma patients according to the expression of D) REST, E) HAR1A, F) HAR1B. Data from the Paediatric Cbioportal (PTBA-provisional, 2182 samples). Gene expression (mRNA) is measured as Z scores, log2 scale. (TIF) [file pone.0312237.s001.tif]

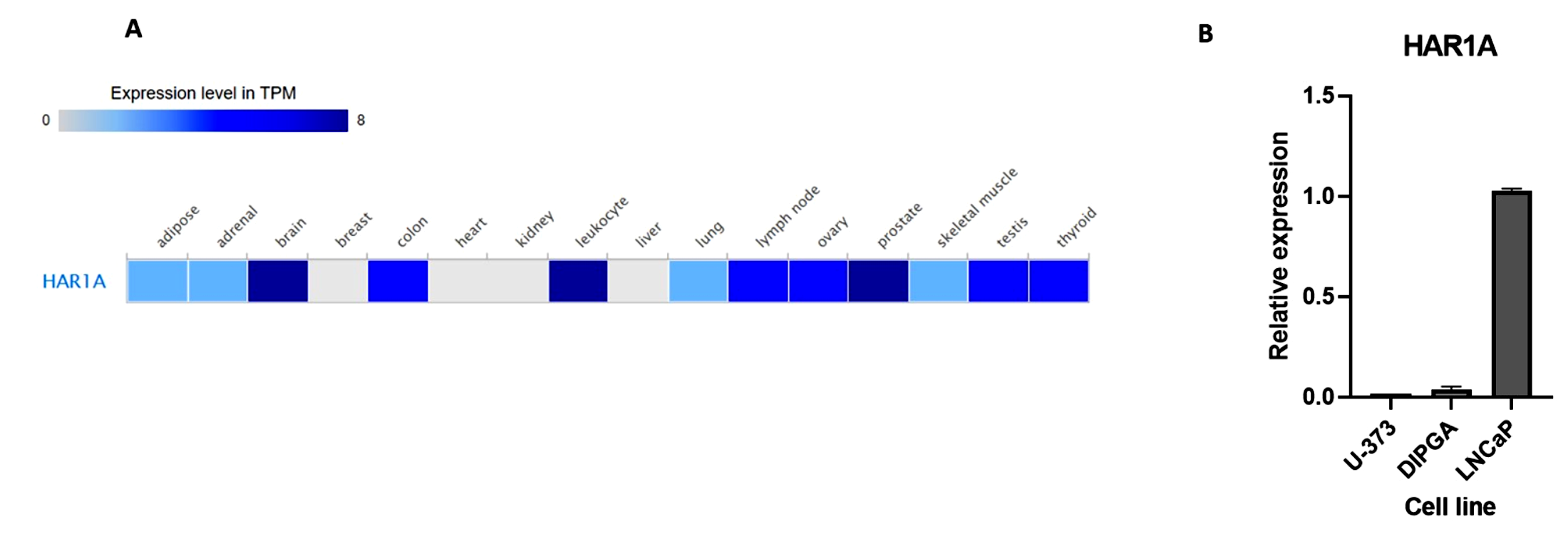

Supplement: S2 Fig — (A) Expression plot of HAR1A from the Illumina Body Map (https://www.ebi.ac.uk/). (B) RT-qPCR (Ct values) of HAR1A expression in LNCaP (prostate), U-373 (brain) and DIPGA (brain) cells. GAPDH was used as reference. (TIF) [file pone.0312237.s002.tif]

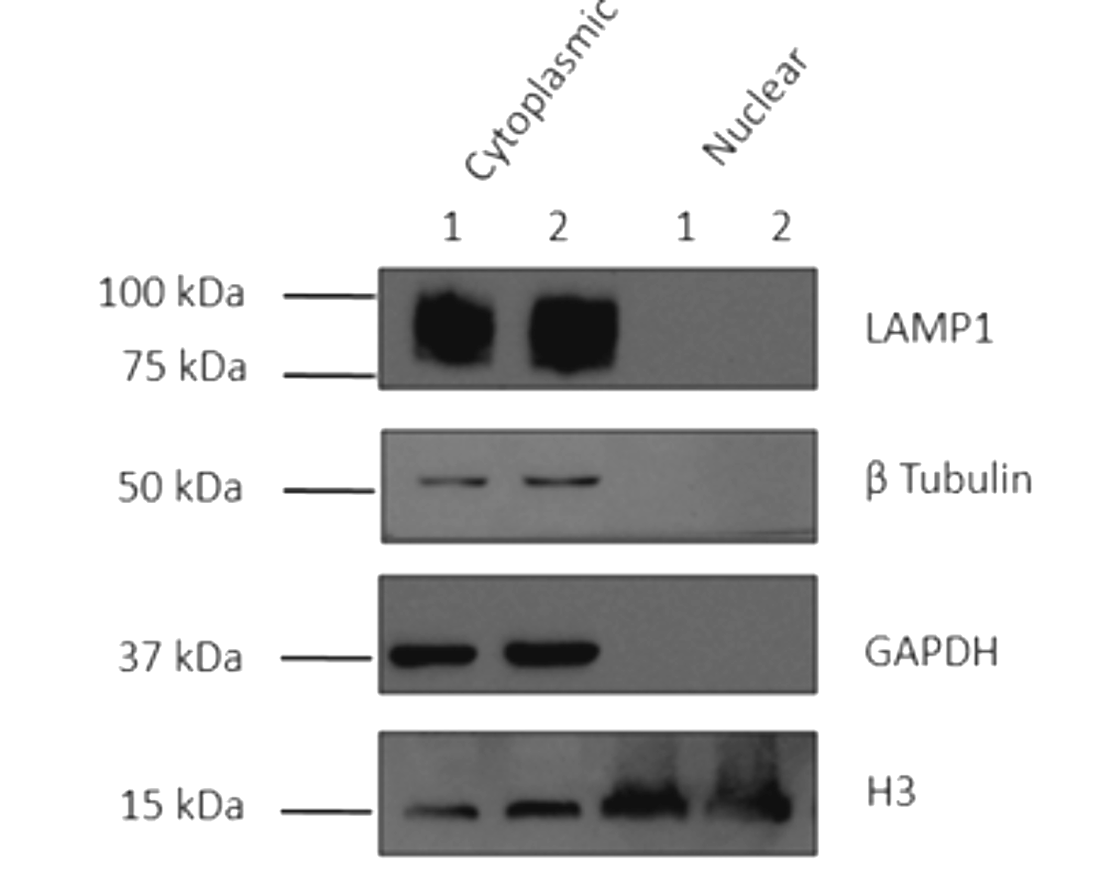

Supplement: S3 Fig — Immunoblotting analysis of preparation of separate nuclear and cytoplasmic lysates from cancer cells trough PARIS Kit protocol (Invitrogen, Cat #AM1921). Cytoplasmic beta-Tubulin (Cell Signaling Cat #2128S) and GAPDH (Cell Signaling Cat #5174T) proteins were adopted to evaluate potential cytosolic contaminations in the nuclear fraction. Lysosomal LAMP1 (Cell Signaling Cat # 9091) protein was used to evaluate potential contamination of organelles in the nuclear fraction. Histone H3 (Invitrogen Cat # 710282) protein was used to confirm that the fraction separated from cytosolic fraction was nuclear. (TIF) [file pone.0312237.s003.tif]
