# Supplementary material for: REST-dependent glioma progression occurs independently of the repression of the long non-coding RNA HAR1A
Source: PLoS One. 2024 Nov 27;19(11):e0312237. doi: 10.1371/journal.pone.0312237 (PMC11602025; doi:10.1371/journal.pone.0312237)
Supplement: S1 Raw images — This Supporting Information file has raw blots showing expression of REST and GAPDH in U-373 cells from Fig 5c; REST and GAPDH in DIPGA cells from Fig 5c; as well raw blots showing expression of LAMP1, beta-tubulin, GAPDH, histone H3 from S3 Fig. (PDF) [file pone.0312237.s004.pdf]

Supporting Information

S4 raw images

U-373

Fig. 5c

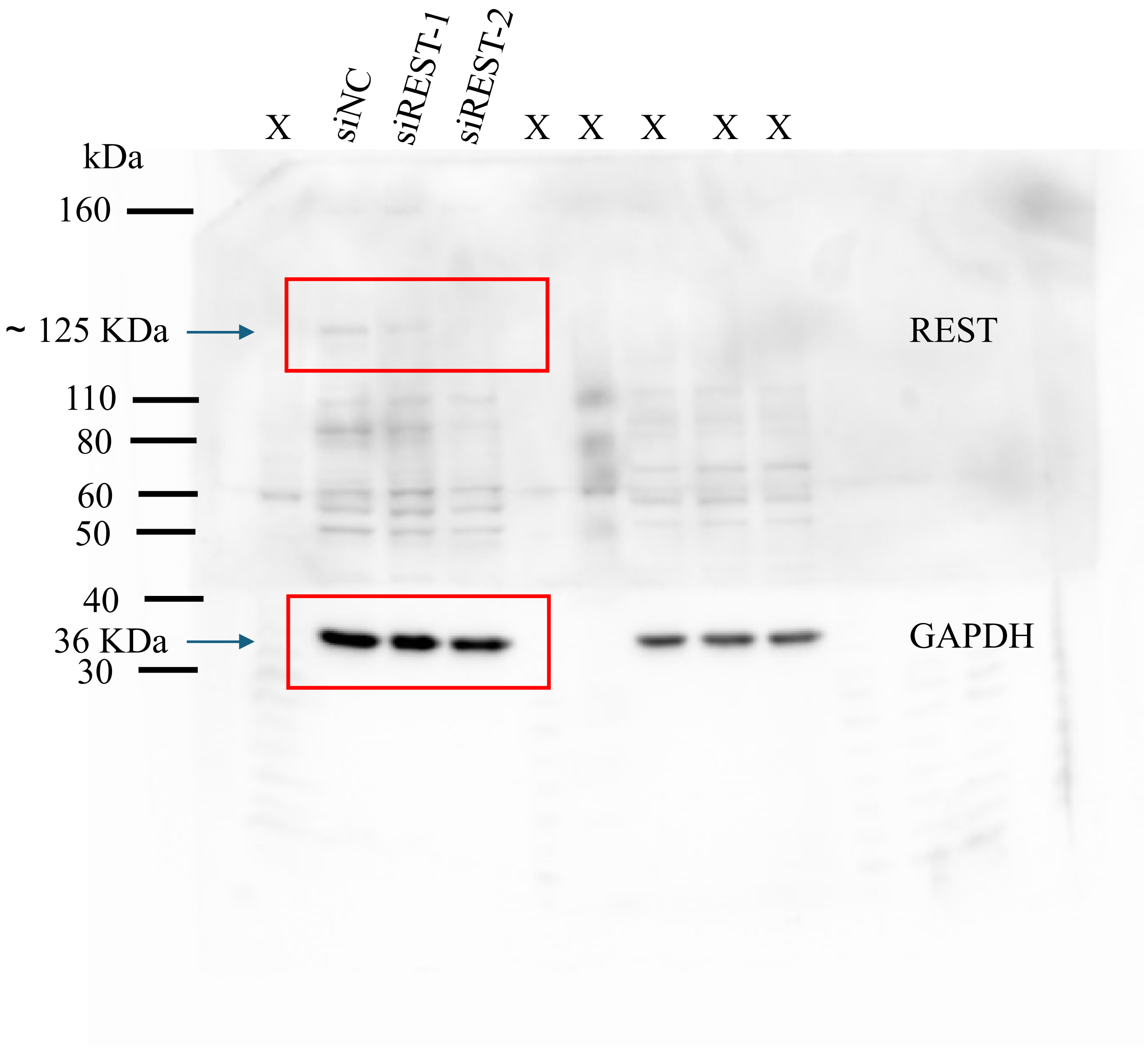

Proteins were visualized using a secondary antibody conjugated to HRP and a chemiluminescence detection system - a digital gel documentation system G:BOX Chemi XX6, Syngene, which captured images of membranes.

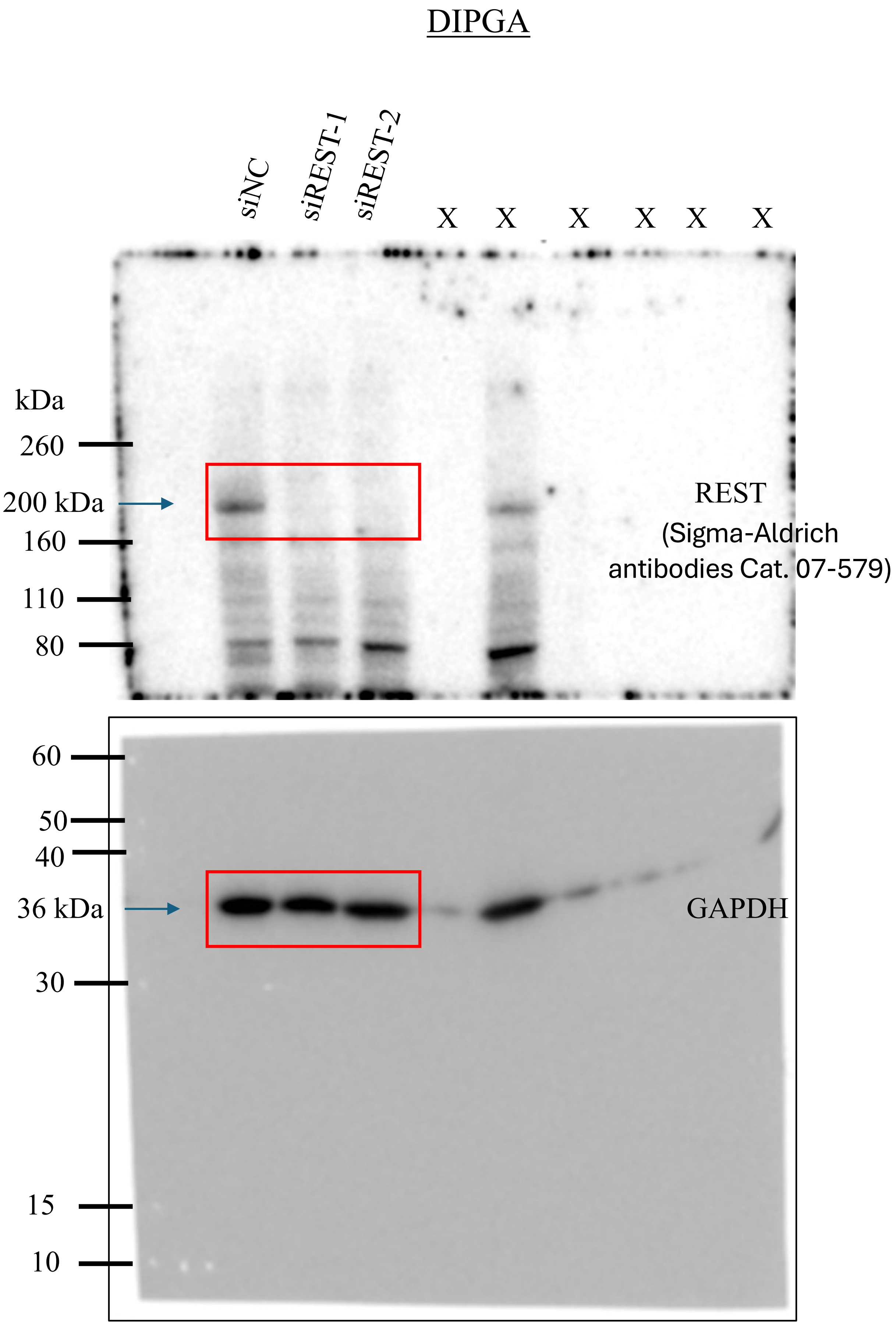

Fig. 5c

Proteins were visualized using a secondary antibody conjugated to HRP and a chemiluminescence detection system - a digital gel documentation system G:BOX Chemi XX6, Syngene, which captured images of membranes.

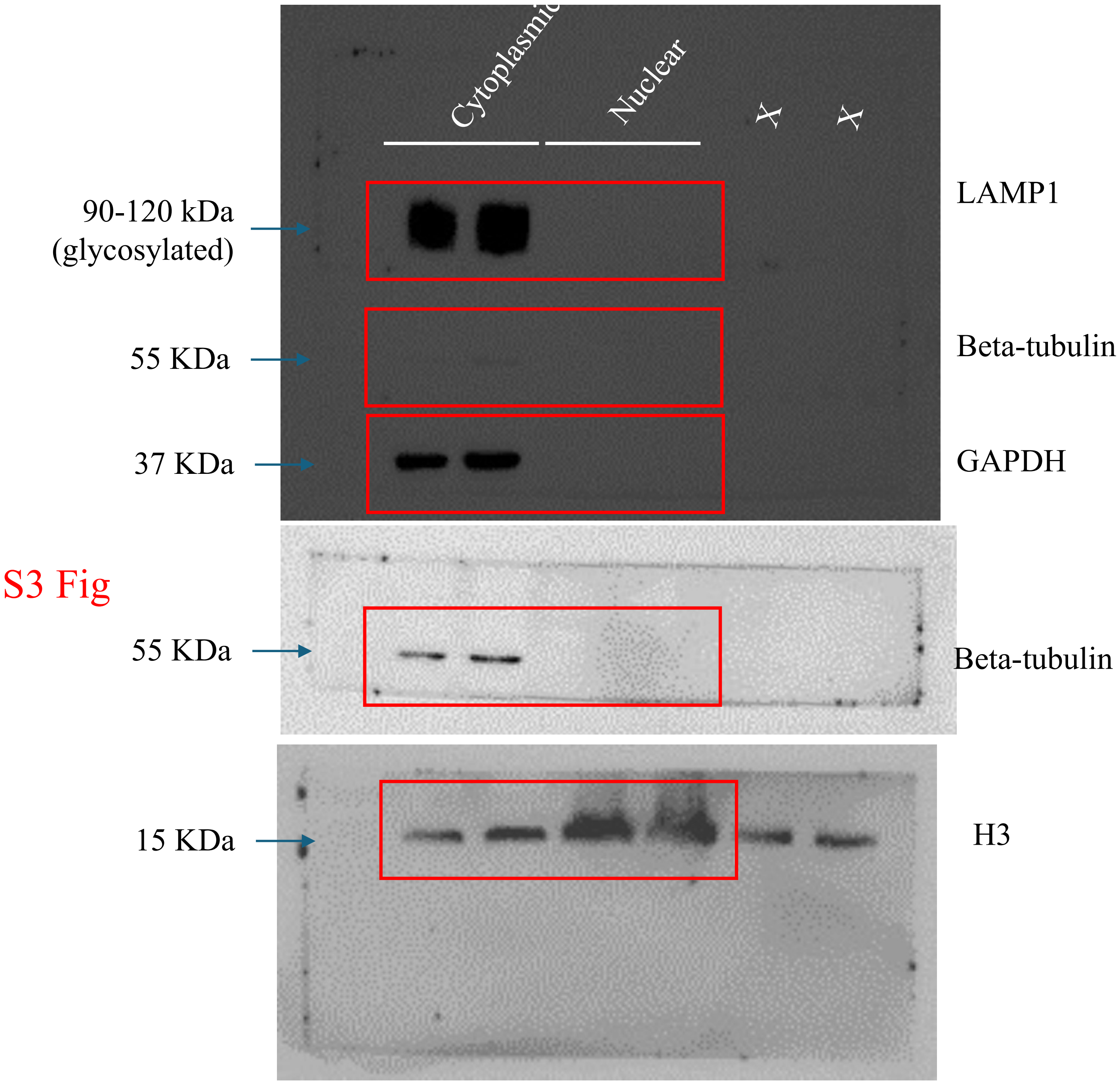

Proteins were visualized using a secondary antibody conjugated to HRP and a chemiluminescence detection system - a digital gel documentation system, ChemiDoc Imaging System, Biorad, which captured images of membranes.
